# Supplementary material for: Subcellular analysis of blood-brain barrier function by micro-impalement of vessels in acute brain slices
Source: Nat Commun. 2023 Jan 30;14:481. doi: 10.1038/s41467-023-36070-6 (PMC9886996; doi:10.1038/s41467-023-36070-6)
Supplement: Supplementary file 1 — Supplementary Information [file 41467_2023_36070_MOESM1_ESM.pdf]

Supplementary Information

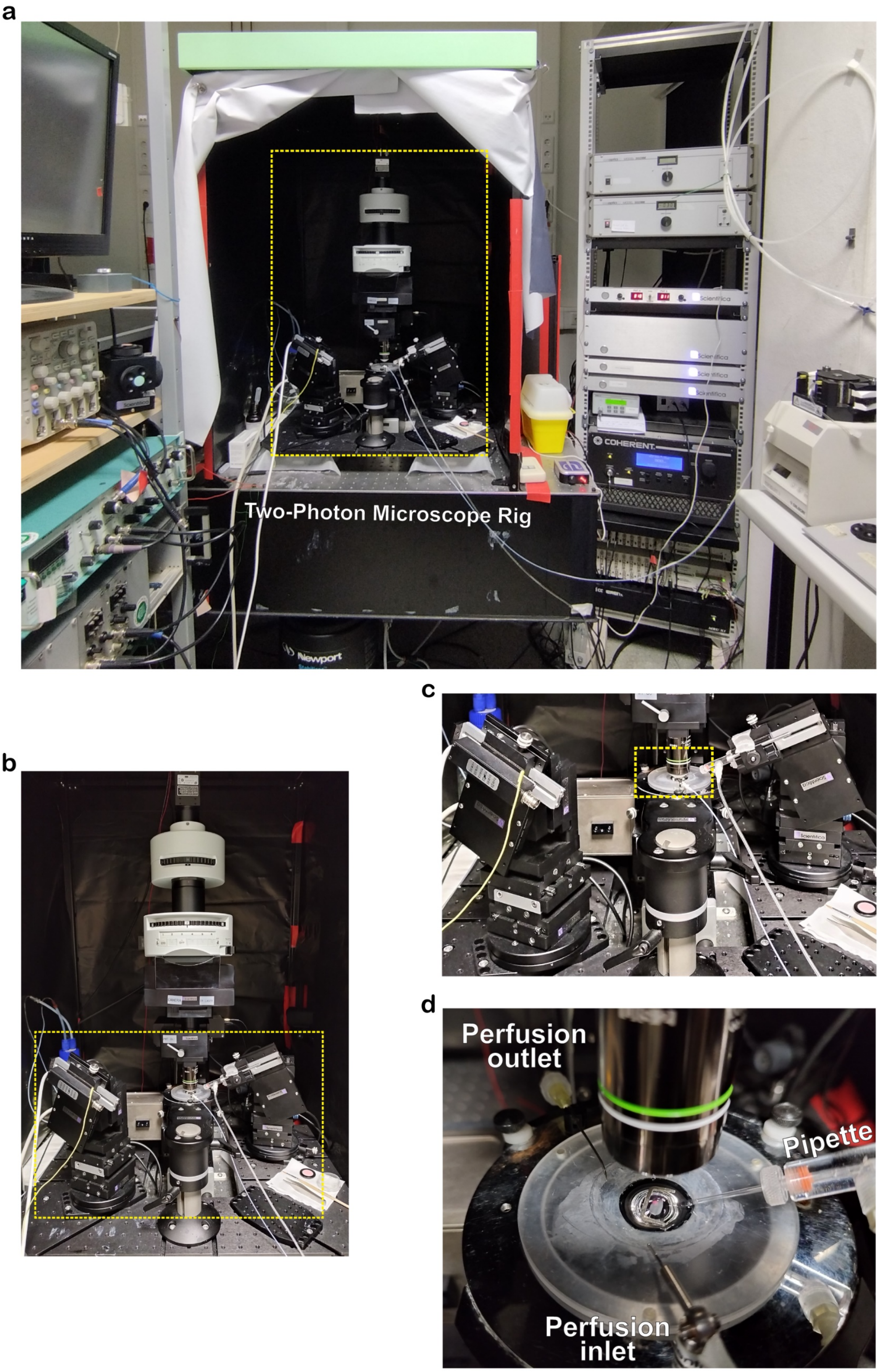

Supplementary Figure 1

Supplementary Fig. 1. The two-photon microscopy setup used alongside the developed in situ BBB model.

a, an overview of the two-photon microscopy setup.

b, the microscopic head and micro-manipulators

c, the setup has 2 micro-manipulators, where the right-hand one was used to guide the pipette.

d, an enlarged view of the glass chamber containing the perfusion buffer and the slice (held down with a metal harp). The pipette is approached using the DIC output monitor.

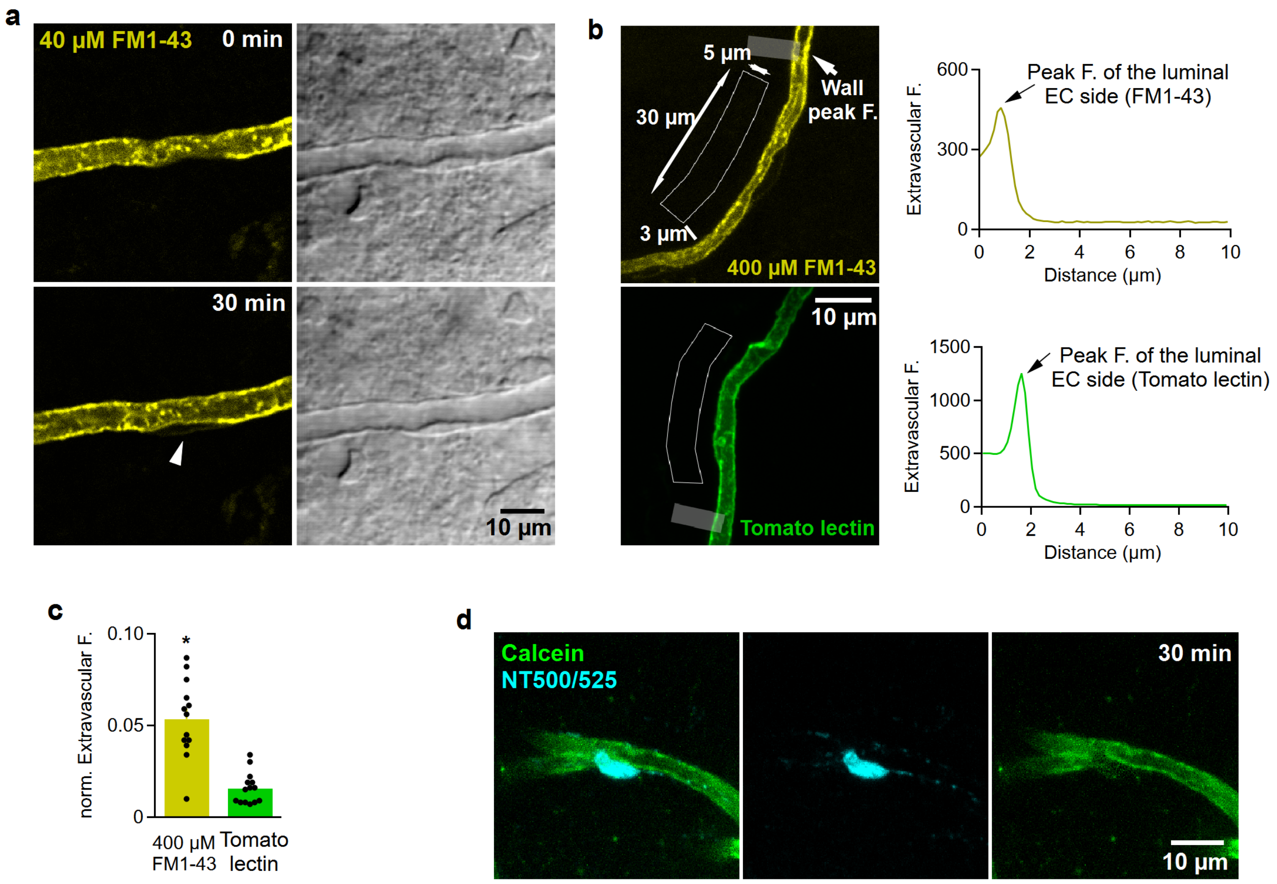

Supplementary Figure 2

Supplementary Fig. 2. Staining of BBB by FM1-43 and NT500/525.

a, The individual fluorescence and Dodt channels at 0 min (top) and 30 min (bottom) showing a capillary with an endothelial cell (EC) (arrowhead) outlined by FM1-43 probe (40 μm). The staining on the abluminal side appears only after 30 min. The images are representative of 3 experiments.

b, The rectangular regions of interest used for the quantification of the dye (FM1-43 or tomato lectin) extravasation and diffusion to the brain parenchyma (see c) are 3 μm away from the capillary, 30 μm in length and 5 μm in thickness. The fluorescence (F.) values were normalized to the average luminal peak fluorescence deduced from the respective intensity line profiles. All measurements were not background corrected. The images are representative of 7 experiments.

c, The diffused FM1-43 dye (at 400 μM) to the brain tissue in the vicinity of capillaries (n_{ROI}=13) was ~3-fold higher than that of tomato lectin (n_{ROI}=14), explaining the staining of the abluminal EC wall in FM1-43 dye but not in tomato lectin assay. Statistical significance was calculated using a two-tailed Student's *t* test (*; P = 0.0001).

d, Pericytes (green) labelled by Neurotrace 500/525 (NT500/525) have a rounded soma protruding from the vessel wall and are separated from the lumen by a layer of tissue, hence distinguished from ECs. Images are representative of 3 experiments.

Source data to panels of Supplementary Figure 2 are provided as a Source Data file. Data are presented as mean values +/- SEM.

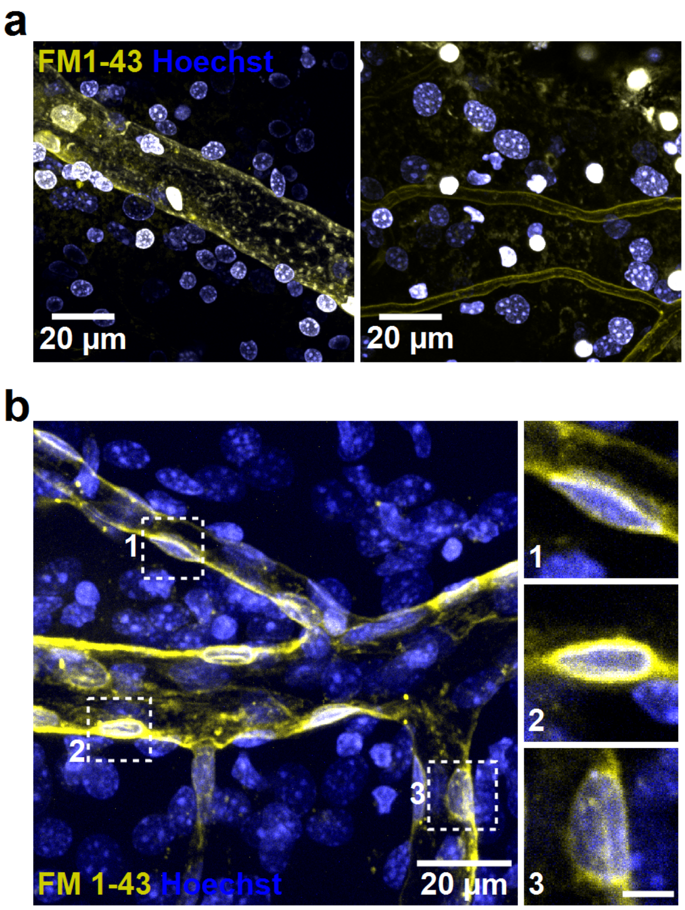

Supplementary Figure 3

Supplementary Fig. 3. Live endothelial cells do not stain with the membrane-permeable DNA stain Hoechst33342 (Hoechst).

a, combining intraluminal FM1-43 application with Hoechst bath-application led to visualizing the outline of endothelial cells and staining the nuclei of neurons and glial cells but not of endothelial cells, suggesting the dye is potently extruded. Note that no nuclei are visible along or in the blood vessel walls (compare to b). Images are representative of at least 4 experiments.

b, after chemical fixation, which inactivates all extrusion systems, Hoechst clearly stained also nuclei of endothelial cells. Scale bar, 5 µm. Images are representative of 4 experiments.
