# Supplementary material for: Subcellular analysis of blood-brain barrier function by micro-impalement of vessels in acute brain slices
Source: Nat Commun. 2023 Jan 30;14:481. doi: 10.1038/s41467-023-36070-6 (PMC9886996; doi:10.1038/s41467-023-36070-6)
Supplement: Supplementary file 3 — Description of Additional Supplementary Files [file 41467_2023_36070_MOESM3_ESM.docx]

**Description of Additional Supplementary Files**

**Supplementary Movie 1:** Guided piercing of a venule’s wall with a custom-made glass pipette and subsequent ejection of a tracer solution. The fast movement of red blood cells away from the injection point indicates a successful piercing and ejection within the branching venules and capillaries.
