# Supplementary material for: Subcellular analysis of blood-brain barrier function by micro-impalement of vessels in acute brain slices
Source: Nat Commun. 2023 Jan 30;14:481. doi: 10.1038/s41467-023-36070-6 (PMC9886996; doi:10.1038/s41467-023-36070-6)
Supplement: Supplementary file 5 — Reporting Summary [file 41467_2023_36070_MOESM5_ESM.pdf]

nature portfolio

Corresponding author(s): Dirk Dietrich

Last updated by author(s): 19/12/22

Reporting Summary

Nature Portfolio wishes to improve the reproducibility of the work that we publish. This form provides structure for consistency and transparency in reporting. For further information on Nature Portfolio policies, see our Editorial Policies and the Editorial Policy Checklist.

Statistics

For all statistical analyses, confirm that the following items are present in the figure legend, table legend, main text, or Methods section.

n/a	Confirmed	
☐	☒	The exact sample size (*n*) for each experimental group/condition, given as a discrete number and unit of measurement
☐	☒	A statement on whether measurements were taken from distinct samples or whether the same sample was measured repeatedly
☐	☒	The statistical test(s) used AND whether they are one- or two-sided<br>*Only common tests should be described solely by name; describe more complex techniques in the Methods section.*
☒	☐	A description of all covariates tested
☐	☒	A description of any assumptions or corrections, such as tests of normality and adjustment for multiple comparisons
☐	☒	A full description of the statistical parameters including central tendency (e.g. means) or other basic estimates (e.g. regression coefficient) AND variation (e.g. standard deviation) or associated estimates of uncertainty (e.g. confidence intervals)
☐	☒	For null hypothesis testing, the test statistic (e.g. *F*, *t*, *r*) with confidence intervals, effect sizes, degrees of freedom and *P* value noted<br>*Give P values as exact values whenever suitable.*
☒	☐	For Bayesian analysis, information on the choice of priors and Markov chain Monte Carlo settings
☒	☐	For hierarchical and complex designs, identification of the appropriate level for tests and full reporting of outcomes
☒	☐	Estimates of effect sizes (e.g. Cohen's *d*, Pearson's *r*), indicating how they were calculated

Our web collection on statistics for biologists contains articles on many of the points above.

Software and code

Policy information about availability of computer code

Data collection	2P scans acquired on Bruker/Scientifica/Nikon platforms with their most recent software version (NIS-Elements AR 4.13.01, Scanimage r3.8, Prairie View 5.4. Confocal image acqusition with NIS-Elements AR 4.13.01.<br>Electrophysiology acquired with Igor Pro (v5-7, Wavemetrics).
Data analysis	Igor Pro (v5-8, Wavemetrics) for graphing and analysis using built-in functions and operations.<br>ImageJ platform (1.51 to 1.53) for ROI and fluorescent profile analysis.<br>LC-MS/MS data output was processed with MaxQuant v1.6.7.0, the heatmap of the percentage change in ABC proteins was generated using Morpheus (https://software.broadinstitute.org/morpheus, v1)

For manuscripts utilizing custom algorithms or software that are central to the research but not yet described in published literature, software must be made available to editors and reviewers. We strongly encourage code deposition in a community repository (e.g. GitHub). See the Nature Portfolio guidelines for submitting code & software for further information.

Data

Policy information about availability of data

All manuscripts must include a data availability statement. This statement should provide the following information, where applicable:

- Accession codes, unique identifiers, or web links for publicly available datasets
- A description of any restrictions on data availability
- For clinical datasets or third party data, please ensure that the statement adheres to our policy

Mass spec data: the annotated spectra and database search output files have been provided and deposited in the public PRIDE server. http://dx.doi.org/10.6019/PXD037454.

Human research participants

Policy information about studies involving human research participants and Sex and Gender in Research.

Reporting on sex and gender	Hippocampal tissue from 5 individuals was analyzed (3 males 27, 61, 21 years old, 2 females 40 and 14 years old).
Population characteristics	See above.
Recruitment	Samples were collected from 5 surgeries in a row.
Ethics oversight	Ethics Committee of the University of Bonn

Note that full information on the approval of the study protocol must also be provided in the manuscript.

Field-specific reporting

Please select the one below that is the best fit for your research. If you are not sure, read the appropriate sections before making your selection.

☒ Life sciences ☐ Behavioural & social sciences ☐ Ecological, evolutionary & environmental sciences

For a reference copy of the document with all sections, see nature.com/documents/nr-reporting-summary-flat.pdf

Life sciences study design

All studies must disclose on these points even when the disclosure is negative.

Sample size	By standard samples are collect from 4, minimum of 3 mice. from each mouse we collect 1-3 slices as indivual samples, depending on the success of the experiments. In this way we minimze the usage of mice and this typically results in sample sizes larger than 4.<br>We arrive at a reasonable n=4 based on the following approximations: with alpha 5% and a power of 80% we are interested in detecting differences between groups which are clearly larger than their variability (STD). In our eyes it is then relatively clear that differences can have a biological meaning. If we want to detect a difference beeing twice larger than the STD with the alpha and power mentioned above we need an n=4 per group, according to n=16(STD/difference)^2 (eg see "Intuitive Biostatisics", Motulsky)
Data exclusions	No data is excluded.
Replication	The minmum number of 4 mice used for every question were prepared on different days, with new solutions etc. So that they represent independent replications. All replications have been included - only experiments which failed technically and could not be analysed were discarded.
Randomization	control and treatment experiments were alternated within the same experimental day to optimize cross-comparability under the same conditions.
Blinding	The experimenter was not blinded against the experimental condition. Effects reported in this study are sufficiently large such that the course of the experiment will reveal the treatment condition. Blinding was therefore not considered to improve data acquisition or analysis.

Reporting for specific materials, systems and methods

We require information from authors about some types of materials, experimental systems and methods used in many studies. Here, indicate whether each material, system or method listed is relevant to your study. If you are not sure if a list item applies to your research, read the appropriate section before selecting a response.

Materials & experimental systems

n/a	Involved in the study	
☐	☒	Antibodies
☒	☐	Eukaryotic cell lines
☒	☐	Palaeontology and archaeology
☐	☒	Animals and other organisms
☒	☐	Clinical data
☒	☐	Dual use research of concern

Methods

n/a	Involved in the study	
☒	☐	ChIP-seq
☒	☐	Flow cytometry
☒	☐	MRI-based neuroimaging

Antibodies

Antibodies used

TMEM119 (rabbit, Abcam, ab209064), VE-cadherin (rabbit, Abcam, ab205336), F4/80 (rat, Novus Biologicals, NB600-404SS). Secondary antibodies: Alexa 488-conjugate (anti-rabbit, Abcam, ab150077), rhodamine red X conjugate (anti-rabbit, Jackson Immune Research Laboratories, 111-295-144), Alexa 568 conjugate (anti rat; Invitrogen, A-11077)

Validation

For each primary and secondary ab negative controls were performed (ommiting that particular ab). Standard antibodies were used with staining patterns known from the literature. As illustrated in the ms the observed staining pattern matched that of published reports. No further validation of primary antibodies was undertaken.

Animals and other research organisms

Policy information about studies involving animals; ARRIVE guidelines recommended for reporting animal research, and Sex and Gender in Research

Laboratory animals

25-35 day old C57black6 mice were used (Charles River).

Wild animals

no wild animals were used.

Reporting on sex

Only male mice were used in this study.

Field-collected samples

No fieled-collected samples were used.

Ethics oversight

All experiments with specimens of murine origin were performed in accordance with the national and institutional guidelines for animal welfare. All procedures were planned and performed in accordance with the guidelines of the University of Bonn Medical Centre Animal-Care-Committee.

Note that full information on the approval of the study protocol must also be provided in the manuscript.
